# Excited state dynamics and exciton diffusion in triphenylamine/dicyanovinyl push–pull small molecule for organic optoelectronics

**DOI:** 10.1038/s41598-020-78197-2

**Published:** 2020-12-03

**Authors:** Benedito A. L. Raul, Yuriy N. Luponosov, Wenyan Yang, Nikolay M. Surin, Olivier Douhéret, Jie Min, Thomas L. C. Jansen, Sergei A. Ponomarenko, Maxim S. Pshenichnikov

**Affiliations:** 1 Zernike Institute for Advanced Materials, University of Groningen, Nijenborgh 4, 9747 AG Groningen, the Netherlands; 2 Enikolopov Institute of Synthetic Polymeric Materials of the Russian Academy of Sciences, Profsoyuznaya 70, Moscow, 117393 Russia; 3 Chemistry Department, Moscow State University, 1/3 Leninskie Gory, Moscow, 119991 Russia; 4 The Institute for Advanced Studies, Wuhan University, Wuhan City, 430072 Hubei Province China; 5 Materia Nova R&D Center, Avenue Nicolas Copernic 3, 7000 Mons, Belgium; 6 Key Laboratory of Materials Processing and Mold (Zhengzhou University), Ministry of Education, Zhengzhou, 450002 China

**Keywords:** Excited states, Optical spectroscopy, Chemical physics, Fluorescence spectroscopy, Solar cells, Density functional theory

## Abstract

Triphenylamine-based small push–pull molecules have recently attracted substantial research attention due to their unique optoelectronic properties. Here, we investigate the excited state de-excitation dynamics and exciton diffusion in TPA-T-DCV-Ph-F small molecule, having simple chemical structure with asymmetrical architecture and end-capped with electron-withdrawing *p*-fluorodicyanovinyl group. The excited state lifetime in diluted solutions (0.04 ns in toluene and 0.4 ns in chloroform) are found to be surprisingly shorter compared to the solid state (3 ns in PMMA matrix). Time-dependent density functional theory indicates that this behavior originates from non-radiative relaxation of the excited state through a conical intersection between the ground and singlet excited state potential energy surfaces. Exciton diffusion length of ~ 16 nm in solution processed films was retrieved by employing time-resolved photoluminescence volume quenching measurements with Monte Carlo simulations. As means of investigating the device performance of TPA-T-DCV-Ph-F, we manufactured solution and vacuum processed bulk heterojunction solar cells that yielded efficiencies of ~ 1.5% and ~ 3.7%, respectively. Our findings demonstrate that the short lifetime in solutions does not hinder per se long exciton diffusion length in films thereby granting applications of TPA-T-DCV-Ph-F and similar push–pull molecules in vacuum and solution processable devices.

## Introduction

Conjugated small molecules, by virtue of their well-defined molecular structure and high reproducibility are deemed attractive for organic optoelectronics applications. Triphenylamine (TPA) based small molecules with the push–pull character have attracted a lot of attention for showing great potential as donor materials in organic solar cells (OSCs)^1,2^ and hole transporting layers in perovskite solar cells^3^. Among them, asymmetrical TPA/dicyanovinyl (DCV) based π-conjugated push–pull small molecules can be considered as one of the simplest class of donor materials for OSCs^4,5^.

TPA/DCV based compounds offer many attractive advantages over polymers counterparts, such as vacuum and solution processability, facile synthesis and purification which allows for flexibility in molecular design, and better batch-to-batch reproducibility^6–8^. Kozlov et al*.* have demonstrated that such materials provide the possibility of long exciton diffusion length^9^, while their push–pull character facilitates efficient intra- and intermolecular charge separation^10^ which potentially might lead to high values of open-circuit voltage^9^. The aforementioned properties make TPA/DCV based materials promising candidates for commercial scale applications.

Despite the extensive research on TPA/DCV small molecules, there are just few experimental and theoretical works which investigate their excited state photophysical behavior^6,10–15^. Specifically, studies geared towards understanding the ultrafast photo-induced processes in solution and the corresponding correlation to the solid-state have not been thoroughly addressed. Therefore, a detailed knowledge about the underlying mechanism governing the excited state dynamics in such materials is of utmost research importance to further improve the molecular design and push the frontier of device performance to the next level^16–18^.

In this paper, we report on the excited state relaxation pathways and exciton diffusion dynamics in a TPA/DCV based small molecule (TPA-T-DCV-Ph-F). We show that the excited state lifetime in solutions amounts to ~ 0.04 ns in toluene and ~ 0.4 ns in chloroform, which is substantially shorter than ~ 3 ns in the PMMA matrix. This unusual behavior is assigned to excited state non-radiative depopulation via conical intersection between the ground and excited state potential energy surfaces. By combining time-resolved photoluminescence (PL) volume quenching experiments with Monte-Carlo simulations, the exciton diffusion length in solution processed films of TPA-T-DCV-Ph-F is obtained as ~ 16 nm and found to be limited by the energetic disorder. To study the potential of TPA-T-DCV-Ph-F as a donor material in OSCs, vacuum and solution processed solar cells were manufactured with 3.7% and 1.5% power conversion efficiencies (PCE), respectively. The obtained results suggest that the TPA-T-DCV-Ph-F molecule offers promising properties for organic optoelectronics provided that the energetic disorder is reduced.

## Results and discussion

### Synthesis, solubility, thermal stability, and electrochemical properties

Fluorination of chemical blocks in conjugated systems is regarded as a promising tool to control intramolecular and intermolecular interactions^19–23^. The phase behavior, glass transition temperature and crystallinity of conjugated molecules can be precisely tuned by adjusting the number and positions of fluorine atoms. Furthermore, incorporating fluorine atoms into the chemical structure can effectively modulate the optical and optoelectronic properties, and therefore affect the device performance.

**Figure 1:**
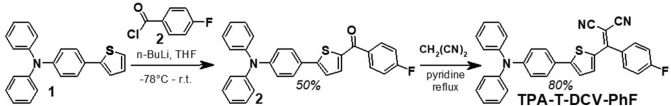
Synthetic route to TPA-T-DCV-Ph-F.

The synthetic route to TPA-T-DCV-Ph-F (Fig. 1) consists of two steps and is similar to its non-fluorinated analog TPA-T-DCV-Ph^9^. In the first step, {5-[4-(diphenylamino)phenyl]-2-thienyl}(4-fluorophenyl)methanone (**2**) was prepared in a reaction between lithium derivative of diphenyl[4-(2-thienyl)phenyl]amine^9^ (**1**) and 4-fluorobenzoyl chloride in 50% yield. In the final second step, TPA-T-DCV-Ph-F was obtained in 80% yield by Knӧvenagel condensation between ketone (**2**) and malononitrile in pyridine using a microwave heating.

TPA-T-DCV-Ph-F has good solubility in chloroform (56 g/L) and in commonly used organic solvents, such as toluene, tetrahydrofuran and chlorobenzene; making the compound highly attractive for solution processed organic optoelectronics.

TPA-T-DCV-Ph-F exhibits excellent thermal stability both in air and under nitrogen with high decomposition temperatures at 389 °C and 397 °C (ESI, Sect. 1.2) respectively, which is similar to that of its analog TPA-T-DCV-Ph^9^. The differential scanning calorimetry (DSC) studies of TPA-T-DCV-Ph-F revealed that the introduction of fluorine into the structure of the molecule influences the phase behavior. The first heating DSC scan (ESI, Sect. 1.2) shows high values of melting temperature (T_m_) but significantly lower value of melting enthalpy (Δ*H*_m_) compared to its non-fluorinated analog TPA-T-DCV-Ph^9^. This indicates that the fluorinated phenyl weakens intermolecular interactions that affect the molecular packing as it was previously reported for other fluorinated conjugated materials^23–25^. However, after having melted TPA-T-DCV-Ph-F becomes amorphous as is evidenced by the subsequent second heating DSC scan, showing only a glass transition temperature (*T*_g_) of about 70 °C.

### Excited state dynamics in solution

**Figure 2:**
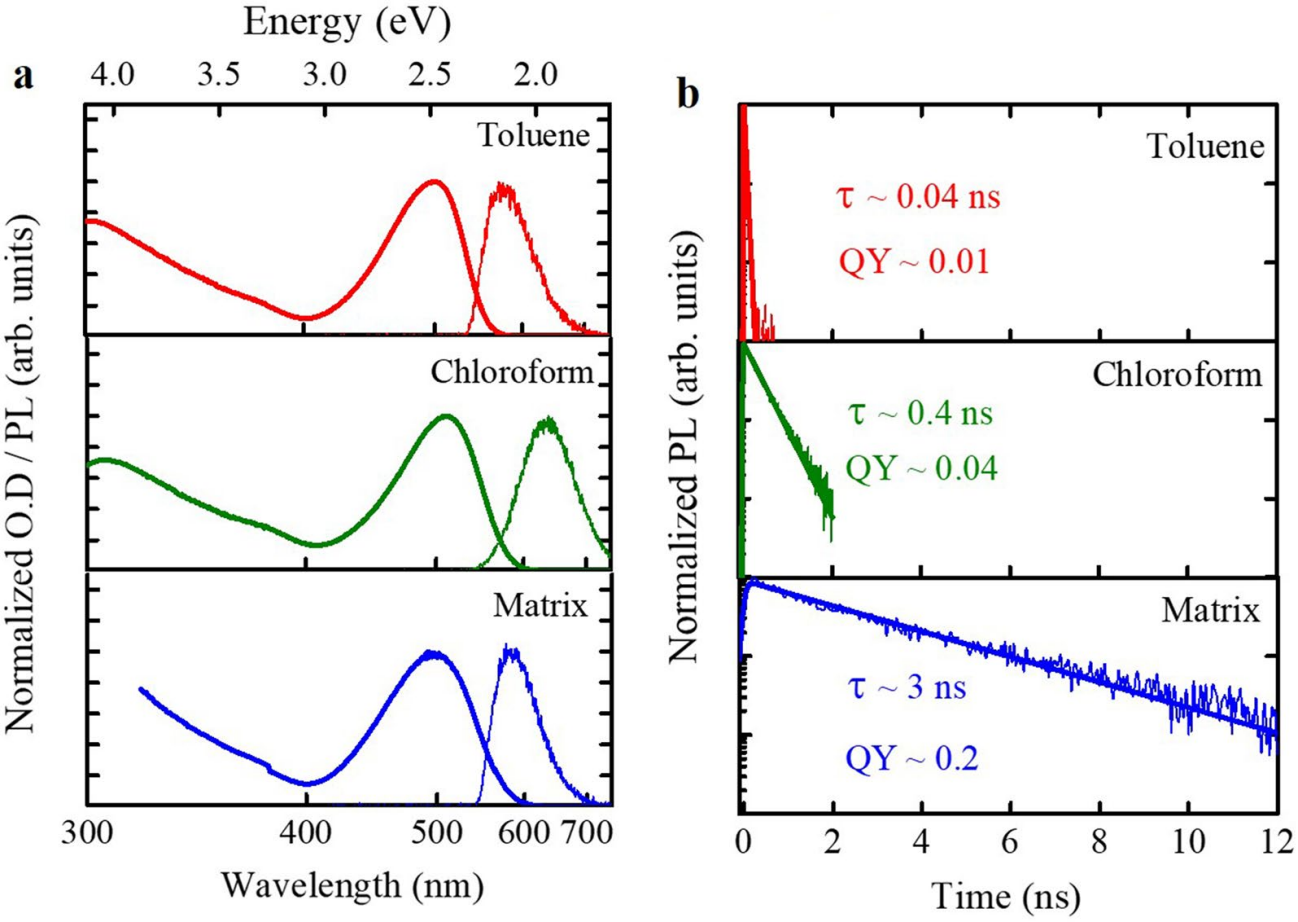
(**a**) Absorption (thick solid lines) and PL spectra (thin solid lines) of TPA-T-DCV-Ph-F in toluene (red) solution, chloroform solution (green), and in PMMA matrix (blue). (**b**) Experimental TPA-T-DCV-Ph-F PL transients (thin solid lines) in toluene (red) solution, chloroform (green) solution and in PMMA matrix (blue), obtained by integrating the PL maps (ESI, Sect. 2) in the 620–700 nm spectral range. The thick solid lines represent the result of mono-exponential fitting. The decay times and respective PL QY’s are shown next to the transients.

The absorption and PL spectra of TPA-T-DCV-Ph-F in diluted solutions (toluene and chloroform) and in poly(methyl methacrylate) (PMMA) matrix are depicted in Fig. 2a. The dispersed TPA-T-DCV-Ph-F molecules in the PMMA matrix is used as a proxy to mimic the solutions with restriction of any potential photo-induced molecular motions, while still preventing intermolecular interactions^10,15^. The absorption spectra consist of two bands (at ~ 300 nm and ~ 500 nm) that are typical for push–pull molecules^11,26^. These bands have been previously assigned as having the mixed character due to the π–π* transition in the conjugated triphenyl-thiophene fragment and intramolecular charge transfer between the electron donating and the electron withdrawing groups^11,26^.

The PL spectra were obtained upon excitation near the absorption maximum (at ~ 520 nm) for optimal light absorption; the Stokes shift values amount to ~ 0.34 eV in toluene and ~ 0.48 eV in chloroform. The larger Stokes shift in chloroform as compared to toluene is ascribed to the higher polarity of the former that leads to increased energy relaxation to longer wavelengths^27,28^. The absorption and PL spectra maxima peak positions of the molecules dispersed in PMMA matrix are slightly shifted compared to the diluted solutions, which is also attributed to the difference in medium polarizability^10,15^.

Figure 2b shows the PL transients of TPA-T-DCV-Ph-F in solutions and in PMMA matrix with the respective independently measured PL quantum yield (QY) values. The PL transients are fitted with mono-exponential function convoluted with a Gaussian apparatus function. The exponential fitting of the PL transients in solution yielded lifetime values of ~ 0.04 ns in toluene and ~ 0.4 ns in chloroform which is in line with increased PL QY from 0.01 to 0.04 in toluene and chloroform, respectively. Both short lifetimes and low PL QYs suggest the prevalence of non-radiative relaxation channels.

TPA-T-DCV-Ph-F undergoes strong change in dipole moment upon photoexcitation, with the electron density in the LUMO mainly delocalized on the acceptor unit DCV-Ph-F and in part on the thiophene linker (ESI, Sect. 3). This causes substantial reorientation of the polar solvent (such as chloroform with the dipole moment of 1.1 D^29^) around TPA-T-DCV-Ph-F molecules^6^; in the less polar toluene (dipole moment of 0.3 D^29^) the effect is much weaker. Therefore, the excited-state lifetimes and the PL QYs appear to be correlated with the solvent polarity. These results are in agreement with earlier studies performed on other dissolved molecules in solvents with descending polarities, in which it was also found that the PL lifetime becomes shorter in less polar solvents^28,30^. It was suggested^28,30^ that the higher dipole moment of the solvent tends to stabilize the conformational changes at the excited state due to stronger solvent-fluorophore dipole–dipole interaction, causing by this manner the reduction in non-radiative decay and the subsequent increase in lifetime.

To demonstrate the applicability of this scenario, we dispersed TPA-T-DCV-Ph-F molecules in a PMMA matrix to restrain the possible molecular conformations^10,15^. This resulted in much longer excited state lifetime of ~ 3 ns with the corresponding increase in PL QY (Fig. 2b). Similar behavior (known as solid state luminescence enhancement) has been previously reported^31,32^ and assigned to a conical intersection (CI) between excited and ground states of the molecule in the liquid phase. To support such scenario for the TPA-T-DCV-Ph-F molecule, we performed time-dependent density functional theory (TDDFT).

### TDDFT calculations

**Figure 3:**
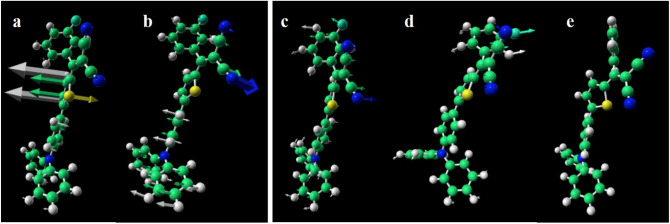
Structures and motion of TPA-T-DCV-Ph-F in the excited states. Two unbound degrees of freedom identified at the excited state at the Frank–Condon point with imaginary frequency of 27i (**a**) and 88i cm^−1^ (**b**). The arrows indicate atomic movements towards the CI. (**c**) The structure of the lowest energy configuration. (**d**) The structure identified near the conical intersection. (**e**) The local minimum configuration on the ground state potential energy surface identified from the energy minimization on the ground state potential energy surface starting from the conical intersection point. The arrows in (**c**) indicate the atomic movement towards the conical intersection structure shown in (**d**), while the arrows in (**d**) indicate the atomic movement towards the local minimum in (**e**). In all panels, the light green atoms are carbon, white is hydrogen, blue is nitrogen, yellow is sulfur and cyan is fluorine. The figure was created using VPython 7 (https://vpython.org).

Electronic structure calculations were performed using the ORCA 4.0.1^33^. We used the ω97X-D3^34^ exchange correlation functional including dispersion in combination with the TZV^35^ basis set. First, the geometry was optimized on the ground state potential energy surface. Then TDDFT^36^ was used to calculate the excited states. The lowest excitation was found at 27,172 cm^−1^ and identified to be the strongest transition. It was verified that the ground state geometry and the lowest excitation energy were not significantly different (< 1% for the excitation energy) than that obtained with the larger and computationally demanding TZVP^36^ basis set. The vibrational frequencies were obtained on both the ground state and the lowest excited state. On the excited state potential, two unbound degrees of freedom were identified. One involves rotation of the thiophene (Fig. 3a), while the other one involves motion of the nitrogen atoms in the DCV moiety as well as rearrangements around the TPA donating group in the other end of the molecule (Fig. 3b). The unbound degrees of freedom on the Franck–Condon point of the excited state surface demonstrate that in the excited state this geometry is not a local minimum, and the molecule can be expected to move when excited^37^. Further analysis is needed to identify the excited state potential minimum.

A geometry optimization on the excited state potential energy surface starting in the ground state minimum configuration (Fig. 3c) deforms the molecule and leads to a geometry where the TDDFT calculations would not converge (Fig. 3d). This is because in this geometry the ground state and lowest excited state are near-degenerate, which leads the TDDFT procedure to fail. We therefore propose that this geometry is close to a CI. From the CI geometry, a further geometry optimization on the ground state potential surface leads to a new ground state geometry (Fig. 3e) with a slightly higher energy than the original geometry. This demonstrates that the molecule has at least one CI and at least two stable configurations on the ground state potential energy surface. The atomic movements (indicated by the arrows) have a large resemblance to the 27i cm^−1^ mode (Fig. 3b) suggesting that this mode indeed plays a crucial role in the photo-isomerization.

**Figure 4:**
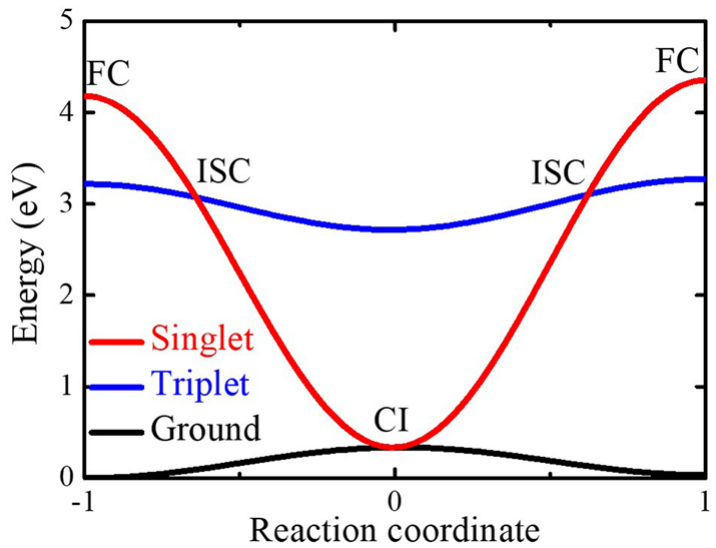
Illustration of the energies along potential energy surfaces. The red, blue and black lines represent the energies of the singlet excited state, the excited triplet, and the ground state, respectively. FC are the Franck–Condon points, CI is the conical intersection position, and ISC represents the singlet–triplet states intersystem crossing positions. The effective reaction coordinate indicates the consecutives excited state configurations.

The illustration of the potential energy surfaces of the lowest electronic states and the ground state as obtained by interpolation between energies obtained for the initial CI, and final geometries is presented in Fig. 4. The energy crossing point is observed between the singlet excited state and the ground state, indicating a non-radiative de-excitation transition at the estimated CI position. The exact energy of the crossing point may be higher as the CI geometry was assigned at the excited state geometry optimization when the TDDFT calculation stopped converging. While excited state relaxations involving CI often happen on sub-picosecond timescales^38^, for the TPA-T-DCV-Ph-F molecule dissolved in toluene, the timescale is ~ 40 ps. In a rigid environment, where the relatively large molecular deformation needed to reach the CI is efficiently suppressed, the relaxation process should be significantly slower, as observed in the experiments above.

Figure 4 also illustrate energy crossing points between the singlet excited state and the lowest triplet state, thereby potentially allowing the intersystem crossing (ISC). We did not explicitly calculate the rates for ISC and CI; however, ISC requires relativistic effects to change the spin while the TPA-T-DCV-Ph-F molecule does not contain heavy atoms. Therefore, the rate for this process is expected to be very low (typically sub-0.1 ns^−1^)^39^ so that the transition through the CI is likely much faster.

### Excited state lifetime and energetic disorder in film

**Figure 5:**
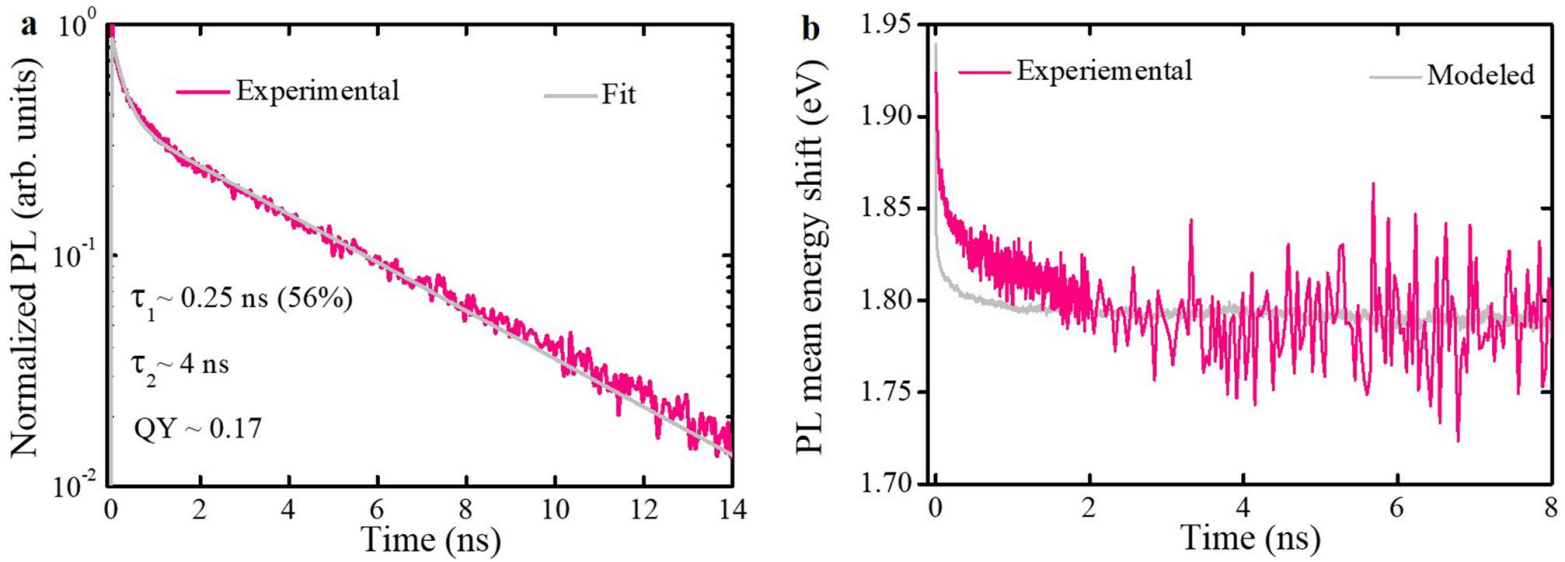
(**a**) PL transient of neat film, was obtained by stitching the fast (< 2 ns) and slow (< 14 ns) transients after the respective integrations of the spectral kinetics in the 550–840 nm range (see ESI, Sect. 4). The solid pink line represents the experimental data, while the solid gray line is the result of bi-exponential fitting. The fitting parameter and PL QY are given next to the transient. (**b**) Experimental (solid pink line) and MC modeled (solid gray line) dynamical shift of PL mean energy. The experimental data was obtained by stitching the fast (< 2 ns) and slower dynamics (< 14 ns). As the density of states in the simulations is centered at 0 eV, we blue-shifted the MC simulations value by 1.95 eV to match the experimental conditions. For clarity, the data is shown up to 8 ns as there are no changes afterwards.

After having established that the excited state lifetime of the isolated TPA-T-DCV-Ph-F molecule varies from 40 ps in toluene solution to 3 ns in a PMMA matrix, we shift to TPA-T-DCV-Ph-F films where conformational movements of the molecule are restricted but intermolecular interactions becomes important. Figure 5a shows the PL transient of TPA-T-DCV-Ph-F solution processed neat film. The bi-modal behavior of the PL transient indicates that several photophysical processes contribute to the excited state dynamics. The fast decay is attributed to intermolecular energy transfer^40^, with contribution of non-radiative decay induced by excitonic traps^41–43^.

The PL transient was fitted with a bi-exponential function convoluted with a Gaussian apparatus function of ~ 10 ps. The average excited state lifetime was determined as:

1 $$\tau_{av} = \frac{{\mathop \sum \nolimits_{i} a_{i} t_{i}^{ 2} }}{{\mathop \sum \nolimits_{i} a_{i} t_{i} }}.$$

where ${a}_{i}$ represent the pre-exponential factor and ${t}_{i}$ is the time constant. The weighted average exciton lifetime was determined to be ~ 3.7 ns while the measured PL QY was ~ 0.17. Both values are similar to those obtained in the PMMA matrix, which further substantiates our previous findings that the photo-induced molecular conformations are mostly restricted in the solid state.

Exciton diffusion in disordered medium is primarily characterized by downhill migration towards lower energy sites, which results on the dynamical red shift of PL^44^. The total shift of PL mean energy stabilizes at $\Delta {E}_{PL}={E}_{PL}\left(t=0\right)-{E}_{PL}(t=\infty )\cong \frac{{\sigma }^{2}}{kT}$, where $\sigma$ stands for standard deviation of the Gaussian density of states (i.e., the energetic disorder). Here the quasi-equilibrium is reached and the thermally activated hopping mainly contributes to the diffusion process^44^. Figure 5b depicts the experimentally tracked dynamical PL mean energy for each time step. The total PL mean energy shift is $\Delta {E}_{PL}\sim 155 \mathrm{meV}$, so that $\sigma \sim 65\mathrm{ meV}$ (at room temperature, $kT \sim 26\mathrm{ meV}$).

### Exciton diffusion length and coefficient

As means of acquiring quantitative information about the temporal and spatial evolution of the excited state population in solution processed films of TPA-T-DCV-Ph-F, we performed time-resolved PL volume quenching experiments combined with MC simulations, out of which the exciton diffusion length and exciton diffusion coefficient were determined^45,46^.

**Figure 6:**
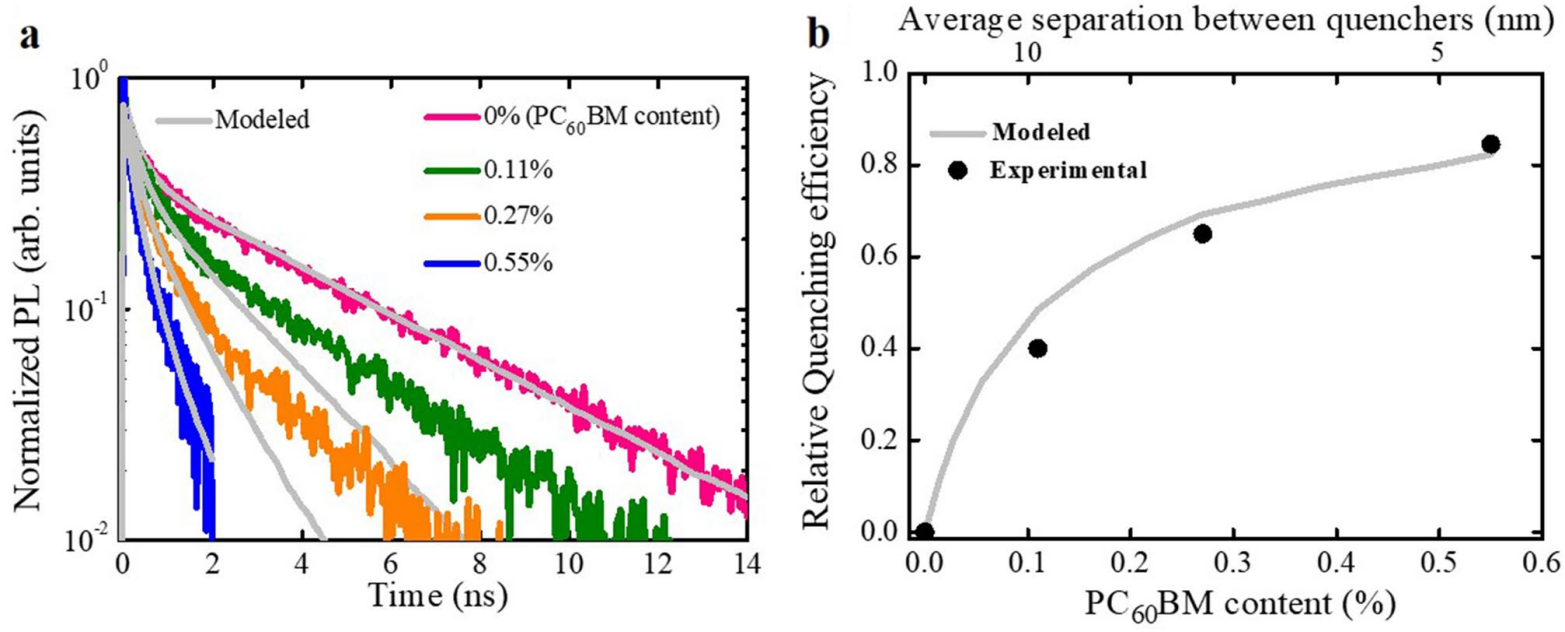
(**a**) Experimental PL transients of neat film and films with different ${\mathrm{PC}}_{60}\mathrm{BM}$ content. Their respective modeled PL transients with MC simulations are depicted in solid gray lines. See ESI, Sect. 6 for all the input parameters. (**b**) Relative quenching efficiency versus ${\mathrm{PC}}_{60}\mathrm{BM}$ content. The black circles are the experimental results and the gray line represent the modeled results. The average separation between ${\mathrm{PC}}_{60}\mathrm{BM}$ molecules was determined as described in ESI, Sect. 5.

The commonly used acceptor in OSCs [6,6]-Phenyl-${C}_{61}$-butyric acid methyl ester (PC_60_BM) was selected as a quenching molecule. The neat film and films mixed with various content of PC_60_BM molecules (0.11%, 0.27% and 0.55% molar ratios) were prepared by spin coating. The respective PL transients are shown in Fig. 6a. As expected, concentration increase of the quencher causes acceleration on the PL transients decay due to the diffusion-limited exciton quenching^45^.

To quantify the quenching process, we plotted the PL quenching efficiency *vs*. ${\mathrm{PC}}_{60}\mathrm{BM}$ content (Fig. 6b). The PL quenching efficiency was determined as^47^:

2 $$Q = 1 - \frac{{\smallint {\text{PL}}_{{{\text{quenched}}}} {\text{d}}t}}{{\smallint {\text{PL}}_{{{\text{neat}}}} {\text{d}}t}}$$

where Q is the PL quenching efficiency, ${\mathrm{PL}}_{\mathrm{quenched}}$ is the PL transient for the samples with different PC_60_BM content, ${\mathrm{PL}}_{\mathrm{neat}}$ is the PL transient for the neat film. Quenching efficiency close to zero is an indication of minimal exciton quenching, which occurs when the average separation of the quenchers is much longer than the exciton diffusion length. Significant quenching sets in when the average separation of the quenchers is shorter than the exciton diffusion length. Therefore, in the one-dimension case excitons diffuse for ~ 10 nm before being quenched, but in three-dimension (3D) there are additional pathways so that the average exciton diffusion length should be longer.

The experimental measured PL transients were modeled using MC simulations of a 3D exciton random walk in a cubic grid (for more details, see ESI, Sect. 6). As the energetic disorder has been obtained earlier, the modeled PL transients (Fig. 6a) were used to extract the exciton hopping time (τ ~ 0.1 ps), which was the only fit parameter. Then the PL quenching efficiencies (Fig. 6b) were calculated using the modeled PL transients. The simulated and experimental results are in good agreement; some discrepancies observed might be attributed to the non-homogenous distribution of $P{C}_{60}BM$ quencher molecules in the films or even possibly due to the formation of small $P{C}_{60}BM$ clusters as reported by Mikhnenko et al.^47^. This discrepancy only affects a small population of the excited state (~ 10%); therefore, it has little effect on the obtained results. The dynamical PL mean energy shift (Fig. 5b) is also satisfactorily described with some inconsistency at early times most probably caused by the limited resolution of the streak-camera.

**Figure 7:**
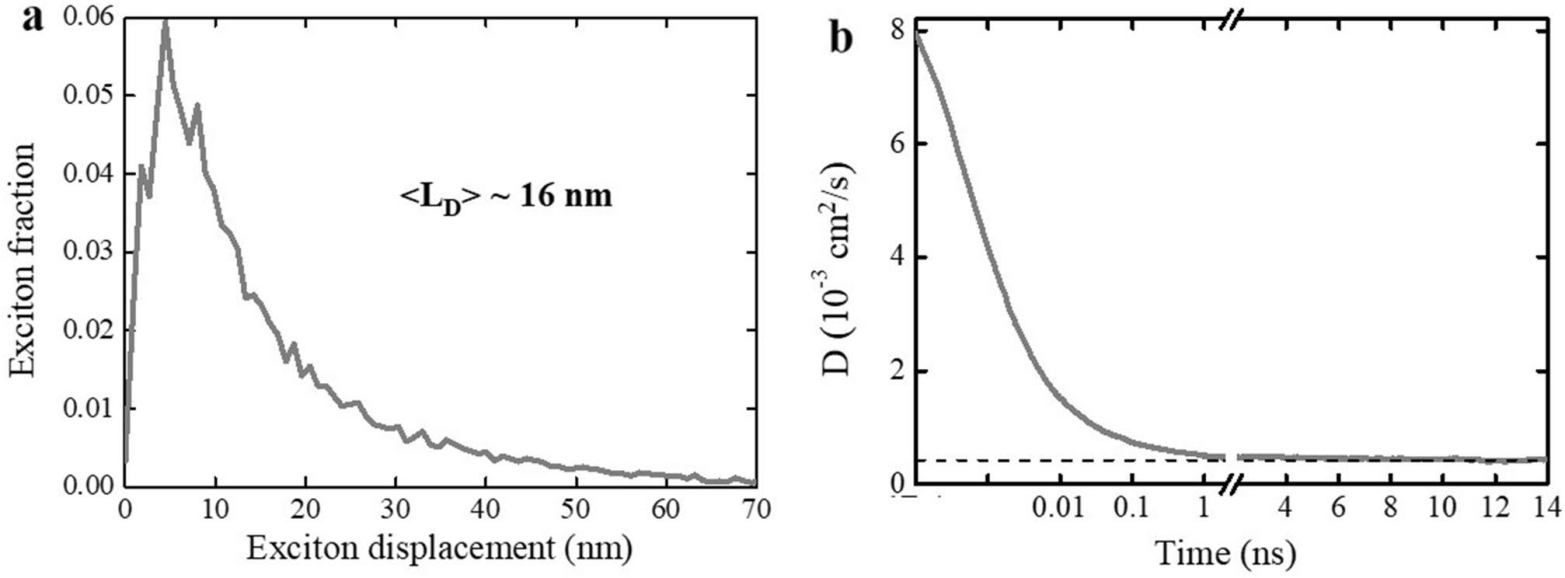
(**a**) Monte-Carlo simulated distribution of exciton displacement in neat thin films of TPA-DVC-Ph-F. (**b**) Time dependent diffusion coefficient. The dash line corresponds to the value where the diffusion coefficient is stabilized at $D \sim 4.2\times {10}^{-4} {\mathrm{cm}}^{2}{\mathrm{s}}^{-1}$. The first 2 ns are presented in the log scale.

The MC simulation data were further used to extract the values of exciton diffusion length and exciton diffusion coefficient. The exciton diffusion length of ~ 16 nm was derived from the exciton displacement statistics as the mean value of displacement (Fig. 7a). In disordered organic semiconductors, exciton diffusion lengths typically range from 5 to 20 nm^13,48,49^; hence, the 16 nm exciton diffusion length in TPA-T-DCV-Ph-F falls into the long range. This is attributed to the long exciton average lifetime of ~ 3.7 ns and the relatively low energetic disorder of ~ 65 meV.

The differential form of the Einstein–Smoluchowski relation (ESI, Sect. 6) was used to obtain the dependence of the exciton diffusion coefficient on time (Fig. 7b). The early times (< 0.1 ns) are dominated by fast exciton cooling followed by slower diffusion where the thermal quasi-equilibrium is reached at the value of diffusion coefficient of $D \sim 4.2\times {10}^{-4} {\mathrm{cm}}^{2}{\mathrm{s}}^{-1}$. For comparison, diffusion coefficient of $D \sim 3.5\times {10}^{-3} {\mathrm{cm}}^{2}{\mathrm{s}}^{-1}$ was obtained in the highly ordered (energetic disorder of < 5 meV) vacuum-deposited C_70_^50^. According to MC simulations, had the energetic disorder been reduced from 65 to 5 meV in solution-processed films of TPA-T-DCV-Ph-F, the exciton diffusion length would have amounted to 50 nm. Therefore, the energetic disorder of ~ 65 meV limits the exciton diffusion coefficient (and the exciton diffusion length) in TPA-T-DCV-Ph-F solution processed films. In order to circumvent this limitation, the energetic disorder should be reduced; this can potentially be achieved by molecular encapsulation^51,52^.

### Device performance

**Figure 8:**
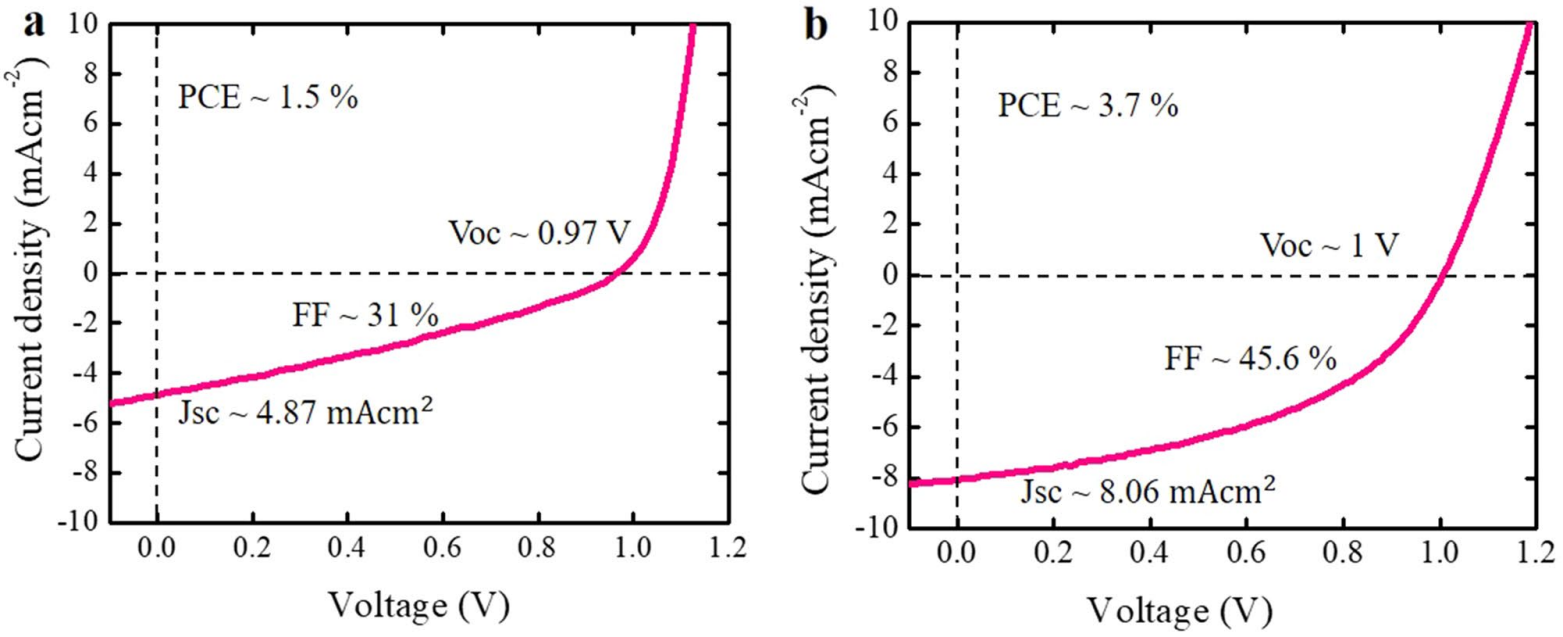
(**a**) Solution processed OSC *J-V* characteristics and the photovoltaic parameters. (**b**) Vacuum processed OSC *J-V* characteristics and the photovoltaic parameters.

To study the potential application of TPA-T-DCV-Ph-F as a donor material in organic solar cells, we fabricated solution processed devices with the conventional structure of indium tin oxide (ITO)/poly(3,4-ethylene dioxythiophene)poly(styrene sulfonate) (PEDOT:PSS)/active layer/perylene diimide functionalized with amino N-oxide (PDINO)/Al^53^, where [6,6]-Phenyl-${C}_{71}$-butyric acid methyl ester (PC_70_BM) was chosen as the electron acceptor material. In order to obtain the best device performances, we carefully optimized the fabrication parameters, including the donor:acceptor weight ratios and doctor-blading speeds (ESI, Sect. 7 for statistics and an AFM surface image). Figure 8a shows the current–density-voltage (*J-V*) curve of the optimized device and the corresponding photovoltaic parameters.

It has been demonstrated that in solution processed bulk heterojunction solar cells with non-optimal morphology, a gradual increase in exciton diffusion length was followed by increase in short-circuit current when the films crystallinity and roughness were improved by meticulous post-deposition treatments^54,55^. Hence, we speculate that the shorter exciton diffusion length of ~ 16 nm in solution processed films of TPA-T-DCV-Ph-F (compared to a longer exciton diffusion length of ~ 25 nm in vacuum evaporated films of its non-fluorinated analog TPA-T-DCV-Ph^9^), limits the device short-circuit current density (*J*_*sc*_) to ~ 4.87 mA cm^−2^. In order to support this supposition, we fabricated vacuum processed bulk heterojunction devices (using the structure ITO/MoO_3_/1:1 TPA-T-DCV-Ph-F: C_70_/bathocuproine/Al) (ESI, Sect. 7 for statistics) and obtained higher *J*_*sc*_ of ~ 8.06 mA cm^−2^ (Fig. 8b), which probably indicates improvements in film morphology. Furthermore, the surface morphology of evaporated pristine TPA-T-DCV-Ph-F thin films, showed an initial bare crystallinity of the material that can be deduced by the observed decrease of the RMS roughness of the surface, from 0.55 nm to 0.32 nm after thermal annealing at 100 °C for 10 min (ESI, Sect. 7 for AFM images). The initial roughness is already extremely low which indicates sharp control of thickness and flatness on evaporated TPA-T-DCV-Ph-F pristine films.

The obtained results demonstrate the potential application of simple TPA-T-DCV-Ph-F molecules for both vacuum and solution processed organic optoelectronics devices.

## Conclusions

In this paper, we have investigated the excited-state and exciton dynamics of a small push–pull molecule TPA-T-DCV-Ph-F. The experimental results revealed that the excited state lifetime in solutions (0.04 ns in toluene and 0.4 ns in chloroform) are shorter than in solid state (3 ns in PMMA matrix). With the support of TDDFT calculations, we concluded that this phenomenon is attributed to excited state non-radiative depopulation in solution from conical intersection between the ground and singlet excited state potential energy surfaces. Furthermore, by combining time-resolved PL and MC simulations we extracted exciton diffusion length of ~ 16 nm in solution-processed layer of TPA-T-DCV-Ph-F. To investigate the potential of TPA-T-DCV-Ph-F as donor material in OSCs, we manufactured vacuum and solution processed solar cells that yielded efficiencies of ~ 3.7% and 1.5%, respectively. Our findings shed light on the photophysics of TPA-T-DCV-Ph-F and pave the way for vacuum and solution processable TPA/DCV based materials with long exciton diffusion length for organic optoelectronic applications.

## Methods

### Sample preparation

The solutions for the neat and mixed thin films were initially separately prepared using chloroform at different concentrations of 10 g/L for TPA-T-DCV-Ph-F and 0.02–0.04 g/L for $P{C}_{60}BM$, then it was let stir for 2 h at room temperature. The solutions were then mixed to achieve the desired $P{C}_{60}BM$ /TPA-T-DCV-Ph-F molar ratios (0.11%, 0.27% and 0.55%) and stirred for another 2 h. The films were prepared by spin coating the solution in a plain glass substrate (1 cm x 1 cm). For the matrix, PMMA (sigma Aldrich, Mw = 120,000 g/mol) was dissolved in chloroform at a concentration of 150 g/L and stirred at room temperature for 8 h. Solution of TPA-T-DCV-Ph-F and PMMA were mixed to achieve the volume ratio TPA-T-DCV-Ph-F/PMMA of 0.2 and stirred for 2 h. The matrix was prepared by spin coating the solution in a plain glass substrate. For the highly diluted toluene and chloroform solutions, the concentrated solutions (10 g/L) were further diluted to obtain optical densities in range of 0.06 to 0.12 at absorption maximum in a 2 mm quartz cuvette.

### Time resolved photoluminescence, absorption and photoluminescence quantum yield

Time-resolved photoluminescence measurements were performed using a streak camera (Hamamatsu, C5680) and a spectrograph. To measure the early time dynamics (< 2 ns), the mode-locked output from the Ti:sapphire oscillator (Mira 900, 76 MHz repetition rate) was focused on the Newport SGC-800 hollow fiber producing a supercontinuum. The excitation wavelength of 520 nm was obtained by selecting a portion of the supercontinuum with a bandpass filter of 520 nm central wavelength and FWHM of 10 nm. For the slower dynamics (< 14 ns), a pulse picker was used to lower the repetition rate of the Ti: sapphire oscillator from 76 to 2 MHz. A long pass filter (OG550) was placed before the spectrograph to filter the excitation stray light. The excitation power was set between 0.5–4 µW to avoid the possibility of exciton-exciton annihilation and photobleaching.

The absorption spectra were obtained with a PerkinElmer Lambda 900 UV/VIS/NIR spectrometer.

The photoluminescence quantum yield of toluene and chloroform solutions were determined by comparing with the known quantum yield of the standard solution of 1,4-bis(5-phenyloxazol-2-yl)benzene (POPOP) in cyclohexane (PL QY ~ 1), using the fluorescence measurement method for optically diluted solutions^56^. Measurements of the photoluminescence quantum yield in PMMA matrix and polycrystalline thin films were carried out using integrating sphere.

## Supplementary information

Supplementary Information
