# Supplementary material for: Excited state dynamics and exciton diffusion in triphenylamine/dicyanovinyl push–pull small molecule for organic optoelectronics
Source: Sci Rep. 2020 Dec 3;10:21198. doi: 10.1038/s41598-020-78197-2 (PMC7713310; doi:10.1038/s41598-020-78197-2)
Supplement: Supplementary file 1 — Supplementary Information [file 41598_2020_78197_MOESM1_ESM.pdf]

Excited State Dynamics and Exciton Diffusion in Triphenylamine/Dicyanovinyl Push-Pull Small Molecule for Organic Optoelectronics

Benedito A. L. Raul[1], Yuriy N. Luponosov[2,3], Wenyan Yang[4], Nikolay M. Surin[2], Olivier Douhéret[5], Jie Min[4,6], Thomas L. C. Jansen[1], Sergei A. Ponomarenko[2,3] & Maxim S. Pshenichnikov[1]*

[1] Zernike Institute for Advanced Materials, University of Groningen, Nijenborgh 4, 9747 AG, Groningen, the Netherlands

[2] Enikolopov Institute of Synthetic Polymeric Materials of the Russian Academy of Sciences, Profsoyuznaya 70, Moscow 117393, Russia

[3] Moscow State University, Chemistry Department,1/3 Leninskie Gory, Moscow, 119991, Russia

[4] The Institute for Advanced Studies, Wuhan University, Wuhan City, Hubei Province, 430072, China

[5] Materia Nova R&D Center, Avenue Nicolas Copernic 3, 7000 Mons, Belgium

[6] Key Laboratory of Materials Processing and Mold (Zhengzhou University), Ministry of Education, Zhengzhou, 450002 China

* Corresponding author

E-mail: m.s.pchenitchnikov@rug.nl

Supplementary information

Section 1. Synthetic procedures……………………………………………………………………….3

Section 1.1. Material characterization………………………………………………………………6

Section 1.2. Solubility, thermal properties, electrochemical properties and photo-stability…………….7

Section 2. Photoluminescence maps and dynamical stokes shift (of solutions and matrix)…………..…8

Section 3. Molecular orbitals calculations……………………………………………………….…9

Section 4. Photoluminescence maps of neat film………………………………………………….10

Section 5. Average separation between quenchers…………….………………………………………10

Section 6. Monte-Carlo Simulations………………………………………………………………11

Section 7. Device fabrication and characterization…………………………………………..…13

References……………………………………..………………………………………………..…16

Section 1. Synthetic procedures

{5-[4-(diphenylamino)phenyl]-2-thienyl}(4-fluorophenyl)methanone (2). 2.5 M solution of *n*-butyllithium (4.77 mL, 10 mmol) in hexane was added dropwise to a solution of compound **1** (2.5 g, 10 mmol) in 70 mL of dry THF at -78 ºC. Afterwards the reaction mixture was stirred for 60 min at -78 ºC and then 4-fluorobenzoyl chloride (1.21 g, 10 mmol) was added in one portion. The reaction mixture was stirred for 1 hour at -78 ºC, then the cooling bath was removed, and the stirring was continued for 1 hour. After completion of the reaction, 150 mL of diethyl ether, 100 mL of water and 5.6 mL of 1 N HCl were added to the reaction mixture. The organic phase was separated, washed with water, dried over sodium sulfate and filtered. The solvent was evaporated in vacuum and the residue was dried at 1 Torr. The product was purified by column chromatography on silica gel (eluent toluene:hexane = 1:1) to give pure compound **2** (1.65 g, 50%) as a green solid. ^{1}H NMR (250 MHz, $CDCl_3$, δ, ppm): 7.04-7.24 (overlapping peaks, 10H), 7.25-7.33 (overlapping peaks, 5H), 7.48-7.59 (overlapping peaks, 3H), 7.85-7.94 (overlapping peaks, 2H). Calcd. (%) for $C_{29}H_{20}NOSF$: C, 77.48; H, 4.48; N, 3.12; S, 7.13; Found: C, 77.57; H, 4.60; N, 3.07; S, 7.04; MALDI-MS: found m/z 449.12; calculated for [M^+] 449.55.

(4-{5-[1,1-dicyano(4-fluorophenyl)methyl]-2-thienyl}phenyl)diphenylamine (TPA-T-DCV-F) Compound **2** (1.08 g, 2.5 mmol), malononitrile (0.50 g, 7.5 mmol) and dry pyridine (24 mL) were placed in a reaction vessel and stirred under argon atmosphere for 22 hours at reflux using the microwave heating. After completeness of the reaction, the pyridine was evaporated in vacuum and the residue was dried at 1 Torr. This crude product was purified by column chromatography on silica gel (eluent dichloromethane:hexane = 1:1). Further purification included precipitation of the product from its THF solution with toluene and hexane to give pure product as a red solid (1.0 g, 80%). M.p.: 227 ºC. ^{1}H NMR (250 MHz, $CDCl_3$): δ [ppm] 7.00 (2H, *J* = 8.9 Hz), 7.07 - 7.15 (overlapping peaks, 6H), 7.17 - 7.24 (overlapping peaks, 2H), 7.26 – 7.33 (overlapping peaks, 5H), 7.43 – 7.50 (overlapping peaks, 4H), 7.64 (d, 1H, *J* = 4.3 Hz). ^{13}C NMR (125 MHz, $CDCl_3$): δ [ppm] 75.30, 114.32, 114.81, 115.96, 116.25, 121.81, 123.66, 124.18, 124.96, 125.36, 127.33, 129.51, 131.71, 131.82, 132.03, 132.08, 135.71, 138.67, 146.64, 149.65, 156.54, 162.79, 162.84, 166.15. Calcd. (%) for $C_{32}H_{20}N_3SF$: C, 77.24; H, 4.05; N, 8.44; S, 6.44. Found: C, 77.32; H, 4.13; N, 8.39; S, 6.38. MALDI-MS: found m/z 497.10; calculated for [M^+] 497.60.

^{1}H and ^{13}C NMR spectra

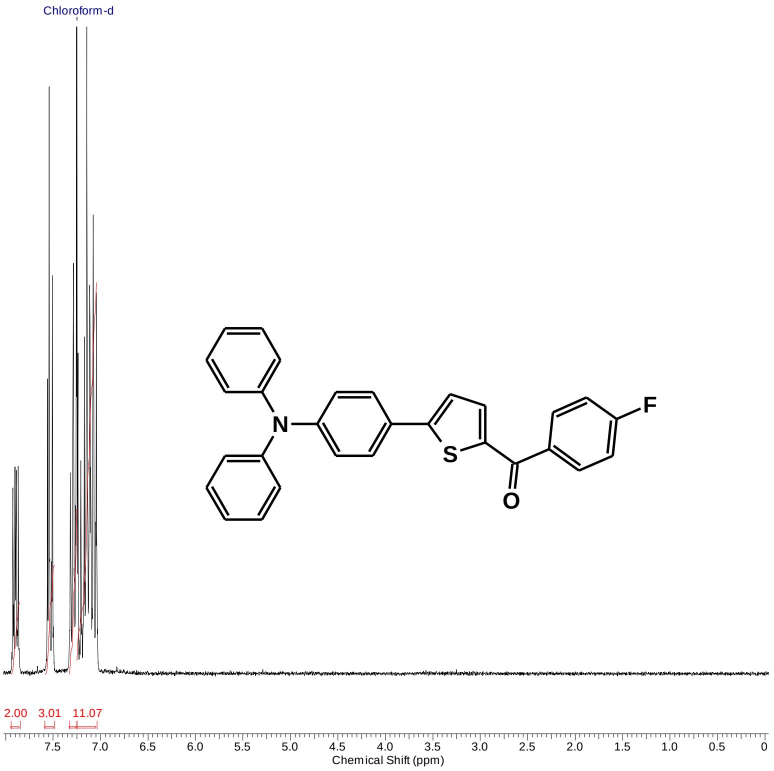

No.	Annotation	(ppm)
1	Chloroform-d	7.25

No.	(ppm)	Value	Absolute Value
1	[7.04 .. 7.25]	11.074	7.61306e+7
2	[7.25 .. 7.33]	5.019	3.45044e+7
3	[7.48 .. 7.59]	3.006	2.06645e+7
4	[7.85 .. 7.94]	2.000	1.37498e+7

Fig. S1 ^{1}H NMR spectrum of compound **2** in $CDCl_3$.

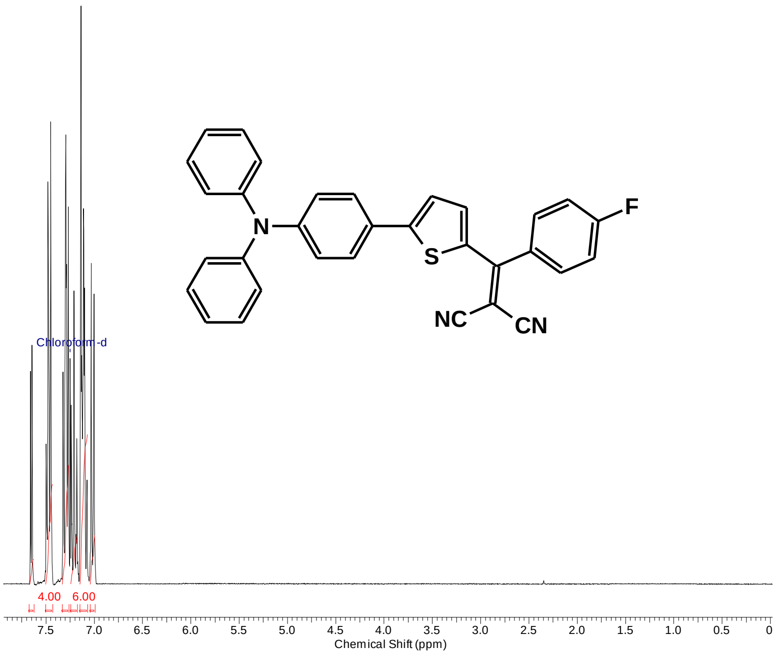

No.	Annotation	(ppm)
1	Chloroform-d	7.25

No.	(ppm)	Value	Absolute Value
1	[6.99 .. 7.04]	2.004	8.42683e+9
2	[7.07 .. 7.15]	6.002	2.52344e+10
3	[7.17 .. 7.24]	2.009	8.44659e+9
4	[7.26 .. 7.33]	5.003	2.10342e+10
5	[7.43 .. 7.50]	3.999	1.68156e+10
6	[7.62 .. 7.67]	1.000	4.20444e+9

Fig. S2 ^{1}H NMR spectrum of TPA-T-DCV-Ph-F in $CDCl_3$.

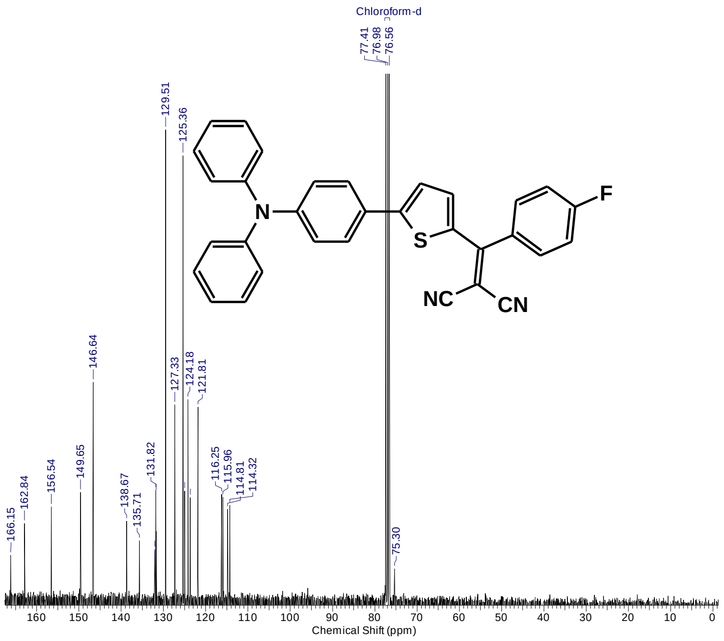

No.	(ppm)	(Hz)	Height
1	75.30	5683.3	0.0457
2	76.56	5778.4	0.9602
3	76.98	5810.4	1.0000
4	77.41	5842.5	0.9703
5	114.32	8628.4	0.1081
6	114.81	8666.0	0.1043
7	115.96	8752.2	0.1161
8	116.25	8774.3	0.1190
9	121.81	9194.2	0.2044
10	123.66	9333.6	0.1155
11	124.18	9372.8	0.2120
12	124.96	9431.4	0.1222
13	125.36	9461.6	0.4513
14	127.33	9610.8	0.2066

No.	(ppm)	(Hz)	Height
15	129.51	9775.3	0.4767
16	131.71	9940.9	0.1204
17	131.82	9949.7	0.1232
18	132.03	9965.5	0.0644
19	132.08	9968.8	0.0537
20	135.71	10243.3	0.0734
21	138.67	10466.4	0.0924
22	146.64	11068.2	0.2289
23	149.65	11295.1	0.1208
24	156.54	11815.1	0.1066
25	162.79	12287.0	0.0678
26	162.84	12290.8	0.0901
27	166.15	12540.4	0.0590

No.	Annotation	(ppm)
1	Chloroform-d	[76.45 .. 77.63]

Fig. S3 ^{13}C NMR spectrum of TPA-T-DCV-Ph-F in $CDCl_3$.

Section 1.1. Material characterization

n-Butyl lithium (2.5 M solution in hexane), 4-fluorobenzoyl chloride, malononitrile were obtained from Sigma-Aldrich Co. and used without further purification. Diphenyl[4-(2-thienyl)phenyl]amine (**1**) was prepared as described in reference[1]. Pyridine, THF, dichloromethane and hexane were dried, purified according to the known techniques and then used as the solvents. All reactions, unless stated otherwise, were carried out under inert atmosphere.

1H NMR spectra were recorded at a Bruker WP-250 SY spectrometer, working at a frequency of 250.13 MHz and using $CDCl_3$ signal (7.25 ppm) and DMSO-d6 (2.50 ppm) as the internal standard. 13C NMR spectra were recorded using a Bruker Avance II 300 spectrometer at 75 MHz. In the case of 1H NMR spectroscopy, the compounds to be analyzed were taken in the form of 1% solutions in $CDCl_3$ or DMSO-d6. In the case of 13C NMR spectroscopy, the compounds to be analyzed were taken in the form of 5% solutions in $CDCl_3$ or DMSO-d6. The spectra were then processed on the computer using the ACD Labs software.

Mass-spectra (MALDI) were registered on the Autoflex II Bruker (resolution FWHM 18000), equipped with a nitrogen laser (work wavelength 337 nm) and time-of-flight mass-detector working in reflections mode. The accelerating voltage was 20 kV. Samples were applied to a polished stainless steel substrate. Spectrum was recorded in the positive ion mode. The resulting spectrum was the sum of 300 spectra obtained at different points of sample. 2,5-Dihydroxybenzoic acid (DHB) (Acros, 99%) and α-cyano-4-hydroxycinnamic acid (HCCA) (Acros, 99%) were used as matrices.

Elemental analysis of C, N and H elements was carried out using CHN automatic analyzer CE 1106 (Italy). The settling titration using $BaCl_2$ was applied to analyze sulfur. Experimental error for elemental analysis is 0.30-0.50%. The Knövenagel condensation was carried out in the microwave “Discovery”, (CEM corporation, USA), using a standard method with the open vessel option, 50 watts. In the case of column chromatography, silica gel 60 (“Merck”) was taken.

DSC analysis of the samples was carried out by a DSC-822e (Mettler-Toledo, Switzerland) at a heating rate 20°C min^{-1} in argon. TGA was done by a Derivatograph-C instrument (MOM, Hungary), at a heating rate 10°C min^{-1} in air and argon.

Solubility of oligomer was measured using its saturated solution in ODCB, which was prepared by stirring of an excess of solid material in the solvent. For this purpose, materials were added in small portions to 1 ml of pure solvent. As prepared, the saturated solutions were filtered through 0.25-mm PTFE syringe filters

and the solvent was evaporated using a rotary evaporator. Afterwards the residue was dried in vacuum at 130°C until it achieved constant weight, which was used to calculate the exact solubility value.

Section 1.2. Solubility, thermal properties, electrochemical properties and photo-stability

Table S1. Solubility and thermal properties of TPA-T-DCV-Ph-F

Compound	Solubility in $CHCl_3$ g L^{-1}	DSC				TGA (T_d/°C)
		T_m/°C	ΔH_m/J g^{-1}	T_g/ °C	ΔS_m J g^{-1} K^{-1}	In air / argon
TPA-T-DCV-Ph-F	56	227	87	70	-0.17	389/397

Notes: T_m - melting temperature; ΔH_m – melting enthalpy; T_g – glass transition temperature; ΔS_m - melting entropy; T_d - decomposition temperature.

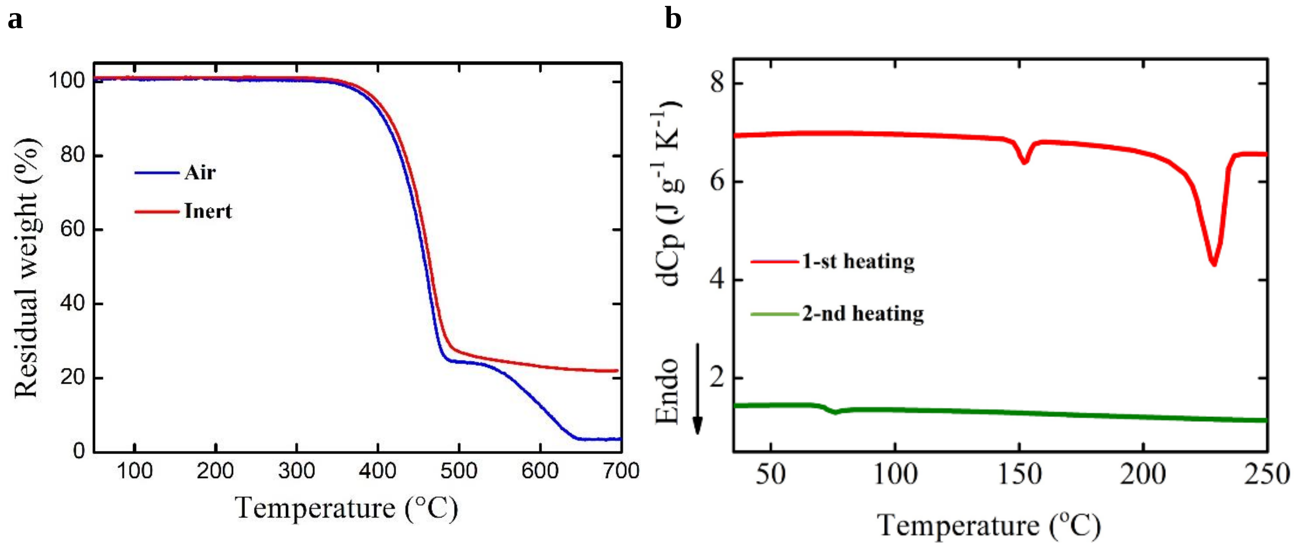

Fig. S4 (a) Thermogravimetric analysis of TPA-T-DCV-Ph-F recorded in air (blue) and under nitrogen (red). **(b)** Differential scanning calorimetry scans of TPA-T-DCV-Ph-F at the first heating (red), cooling, second heating (green).

Section 2. Photoluminescence maps and dynamical stokes shift (of solutions and matrix)

The photoluminescence (PL) maps of TPA-T-DCV-Ph-F in toluene solution, chloroform solution and in PMMA matrix are depicted in Figure S5a, and the respective dynamical Stokes shifts are shown in Figure S5b. The dynamic Stokes shift of 40 meV in toluene is lower compared to the 100 meV in chloroform, which further demonstrates that with the increase in solvent polarity PL shifts to longer wavelengths. In the solid state (i.e., in the PMMA matrix) the dynamic Stokes shift is negligible (~ 0 eV) due to the lack of intermolecular interactions.

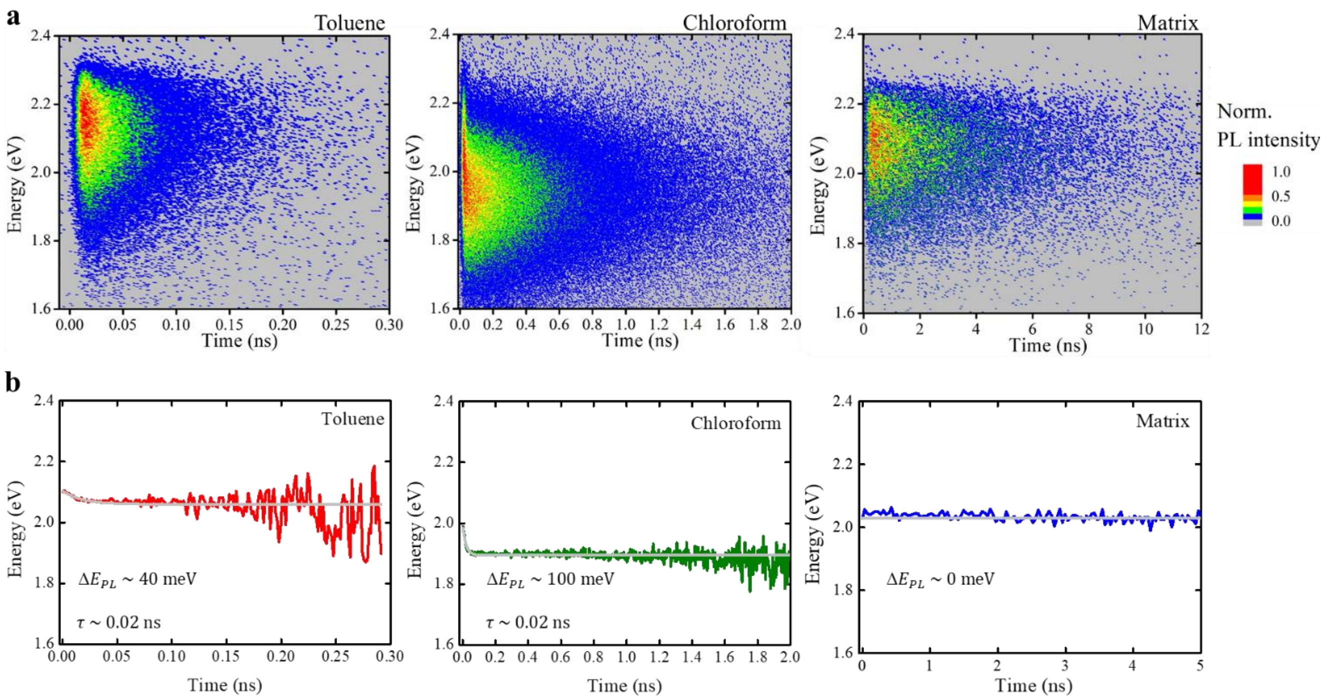

Fig. S5 (a) PL maps of TPA-T-DCV-Ph-F in toluene (left) solution, chloroform solution (middle) and in PMMA matrix (right). The PL maps are normalized by the maximum amplitudes. **(b)** The respective dynamical Stokes shifts calculated as the time-dependence of the mean frequency $<\omega(t)> = \int \omega S(\omega,t)d\omega / \int S(\omega,t)d\omega$ of spectral slices $S(\omega,t)$ at a particular time t. The gray line is the mono-exponential fitting with the fitting values given next to the transients.

Section 3. Molecular orbitals calculations

The HOMO and LUMO levels were calculated in chloroform and toluene using the conductor-like polarizable continuum (CPCM) solvent model[2] at the DFT level with the same exchange correlation functional and basis set as used for the TDDFT calculations (see the main text). Both HOMO and LUMO levels in toluene and chloroform solutions are identical.

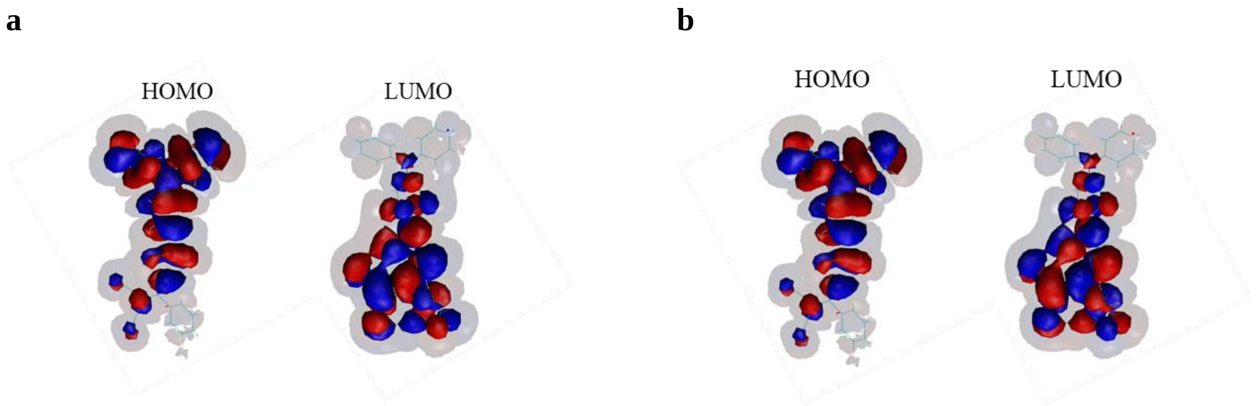

Fig. S6 Representation of electron density of HOMO and LUMO in chloroform solution **(a)** and toluene solution **(b)**. The red and blue orbital isosurfaces represent 0.1% of the maximum values of the positive and negative values, respectively. The gray orbital isosurface is plotted at 0.0125% of maximum value. The calculations were performed using the CPCM solvent model with the standard solvent settings[2]. The sign of the LUMO was arbitrarily set opposite in toluene and chloroform but the sign of the wavefunction has no physical meaning so that the LUMO orbitals are not significantly different.

Section 4. Photoluminescence maps of neat film

Figure S7 shows the stitched PL maps for the fast (< 2 ns) and slower dynamics (< 14 ns). Both PL maps were spectral integrated (in 2.2-1.5 eV range) and the resulting transients were stitched to obtain the final transient presented in the manuscript. The same procedure was applied for all the mixed films.

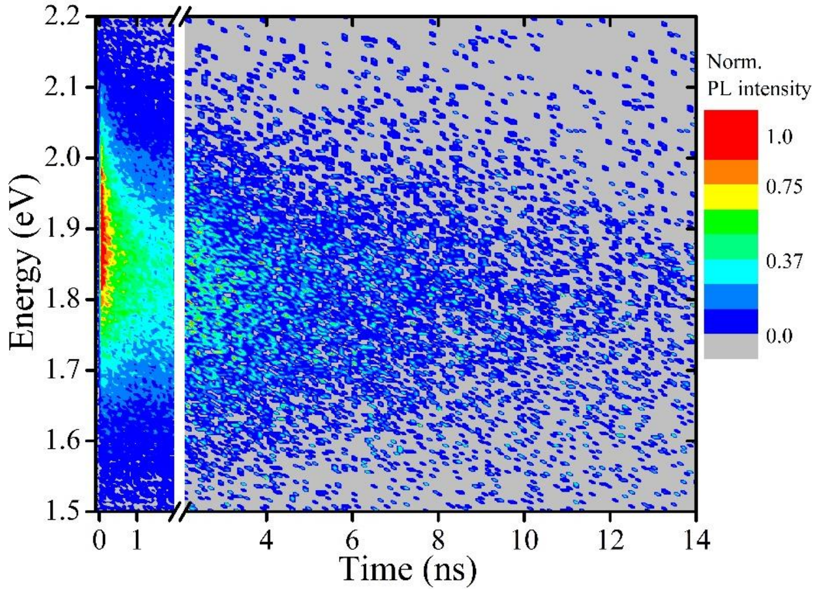

Fig. S7 Stitched PL maps of TPA-T-DCV-Ph-F neat film. The break separates the experimental data obtained at different time bases of the streak-camera.

Section 5. Average separation between quenchers

The average separation between quenchers was calculated as:

$$d = \sqrt[3]{\frac{M_m}{\rho\, N_A\, n_r}} = \sqrt[3]{\frac{1}{n_r}} \times 0.89\ [nm], \qquad (1)$$

where for TPA-T-DCV-Ph-F, ρ ~ 1.17 g⋆cm^{-3} is the density, M_m ~ 497 g⋆mol^{-1} is the molecular mass and 0.89 nm is the size of the molecule. N_A is the Avogadro constant, and n_r is the quencher molar ratio with respect to TPA-T-DCV-Ph-F.

Section 6. Monte-Carlo Simulations

The MC simulations schematics is depicted in Figure S8. The exciton lifetime, hopping distance and quencher content were used as the inputs parameters as shown in Table S2. The distance between two grid points (hopping distance) was equal to the size of the molecule ~ 0.89 nm averaged over three dimensions (see Section 5 for calculations). Each grid point was assigned an energy generated from a Gaussian distribution function centered at zero:

$$g(\varepsilon) = \frac{1}{\sigma\sqrt{2\pi}} e^{-\frac{\varepsilon^2}{2\sigma^2}} \quad (2)$$

where ε is the grid point energy, σ is the energetic disorder (standard deviation)[3].

At the beginning of the simulation, 5000 excitons with a finite lifetime (τ_{av}) were placed randomly at different grids points. The exciton hopping probability p_{if} was set at unity if the initial grid point has an energy (E_i) higher than the final grid point energy (E_f), while for the opposite case ($E_i < E_f$) the probability was determined from a Boltzmann distribution with kT equal to ~ 26 meV (room temperature):

$$p_{if} = e^{-\frac{E_f - E_i}{kT}} \quad E_i < E_f \quad (3).$$

At each time step, every quenched exciton is eliminated from the simulation. The differential form of the Einstein-Smoluchowski relation for random walk was used to obtain the dependence of the exciton diffusion coefficient on time from the known exciton displacement as:

$$D = \frac{\partial < l^2(t) >}{6\partial t} \quad (4)$$

where $< l^2(t) >$ is the average square of the excitons displacement, t is the diffusion time.

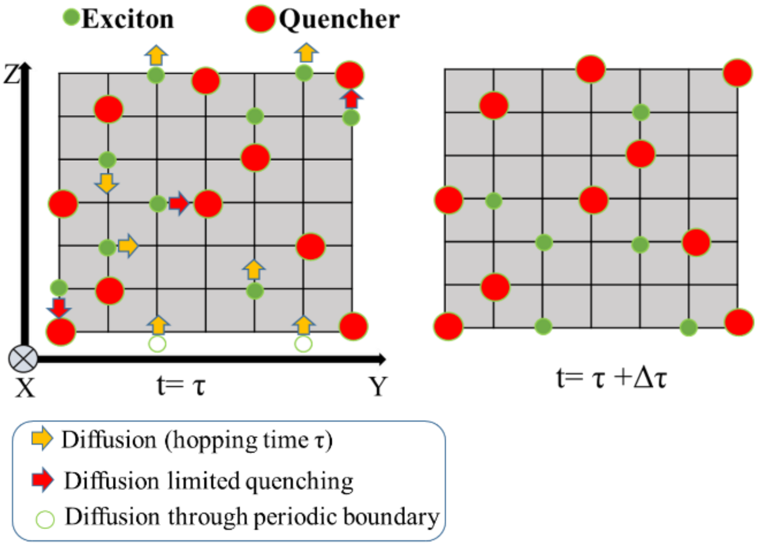

Fig. S8 Schematics of random walk model for Monte-Carlo Simulations. $t = \tau$ is the exciton distribution at time τ. $t = \tau + \Delta\tau$, is the exciton distribution at the next time step.

Table S2. Monte-Carlo Simulations parameters.

Description	Parameter	Value
Cartesian lattice grid	X-Y-Z	500-500-500
Lifetime-1 fraction	a_1	~ 0.55
Lifetime-1	τ_1	~ 0.3 ns
Lifetime-2	τ_2	~ 4.4 ns
Energetic disorder	σ	~ 65 meV
Hopping distance	d	~ 0.89 nm
Quencher fraction	QF	From 0 to 5.26×10^{-3}
Hopping time	τ	~ 0.1 ps

Section 7. Device fabrication and characterization

Solution processed TPA-T-DCV-Ph-F based BHJ OSCs were fabricated with a conventional structure of ITO/PEDOT:PSS/active layer/PDINO/Al. ITO pre-patterned glasses were pre-cleaned for 20 minutes each in an ultrasonic bath with toluene, deionized water, acetone and isopropanol. After drying by fast-blowing high-purity nitrogen, all substrates were cleaned in the UV-ozone cleaning machine for 15 minutes. Afterwards, a ~40 nm PEDOT:PSS thin layer was deposited on the pre-cleaned substrates by spin-coating the PEDOT:PSS aqueous dispersion. Relevant solutions are prepared by stirring in the glovebox overnight. The TPA-T-DCV-Ph-F:PC70BM blends with different weight ratio were fully dissolved in ODCB at a total weight concentration of 20 g/L. The active layer was deposited on the PEDOT:PSS by doctor-blading with different speeds. Subsequently, the PDINO solution was spin-coated onto the active layer. Finally, Al (100 nm) was evaporated onto the PDINO under high vacuum. The J-V curves of all the OSCs were measured under AM 1.5G irradiation provided by an Enli Solar simulator (100 mW cm^{-2}), which was calibrated with a standard single-crystal Si solar cell (made by Enli Technology Co., Ltd., Taiwan. The standard cell has been calibrated by the National Institute of Metrology (NIM), China). EQE spectra were measured by the integrated system (QE-R, Enlitech).

Table S3. Photovoltaic parameters of TPA-T-DCV-Ph-F:$PC_{70}BM$ OSCs under AM 1.5 G illumination at 100 mW cm^{-2}.

Weight ratio D:A	Speed [mm/s]	Voc [V]	Jsc [mA cm^{-2}]	FF [%]	PCE (avg. [a]) [%]
1:0.8	20	1.01	3.05	26.87	0.82 (0.78)
1:0.8	30	1.00	2.83	26.25	0.74 (0.67)
1:0.8	40	1.01	2.17	26.37	0.58 (0.53)
1:0.8	50	1.02	2.54	26.98	0.69 (0.66)
1:0.8	60	1.01	1.29	26.05	0.34 (0.31)
1:1	20	1.00	3.91	28.30	1.11 (1.09)
1:1	30	0.99	3.22	27.97	0.89 (0.87)
1:1	40	1.00	2.24	26.03	0.59 (0.55)
1:1	50	0.99	1.36	26.79	0.36 (0.33)
1:1	60	1.00	1.47	25.89	0.38 (0.34)
1:2	20	0.96	3.22	29.04	0.90 (0.84)

1:2	30	0.96	2.73	27.14	0.71 (0.65)
1:2	40	0.97	3.10	27.76	0.83 (0.79)
1:2	50	0.95	4.80	29.54	1.34 (1.31)
**1:2**	**60**	**0.97**	**4.87**	**31.11**	**1.46 (1.42)**
1:3	20	0.91	4.74	31.19	1.35 (1.33)
1:3	30	0.92	4.04	29.26	1.09 (1.03)
1:3	40	0.93	3.11	28.00	0.81 (0.77)
1:3	50	0.93	2.56	27.51	0.66 (0.64)
1:3	60	0.93	2.63	26.90	0.65 (0.62)
1:4	40	0.92	4.20	27.64	1.07 (1.01)
1:4	50	0.93	4.11	29.01	1.11 (1.00)
1:4	60	0.94	4.48	29.41	1.23 (1.17)
1:4	70	0.93	3.73	28.14	0.97 (0.89)

[a] The average value in bracket are obtained from 12 devices.

Vacuum processed BHJ OSCs based on TPA-T-DCV-Ph-F were fabricated as follow. Prior to deposition the substrates were cleaned with 1:10 RBS 25 concentrate (Chemical products R. Borghgraef S.A.): Di-ionized (DI) water, then rinsed with tap water and DI water and dried with N_2. 10 min exposure to O_2 plasma using a Plasma Cleaner (Harrick Plasma) helps to improve the adhesion of the first barrier layer on the anodic substrate. The layers composing the photovoltaic stack were deposited by thermal sublimation (see below for technical specifications).

The photovoltaic devices consisted of a ITO/glass substrate on which a 25 nm-thick MoO_3 barrier layer, a 100 nm-thick 1:1 (wt%) TPA-T-DCV-Ph-F:C_{70} photoactive layer, 10 nm of BCP (bathocuproine as exciton-blocking layer) and a 100 nm-thick Al cathode were successively deposited. When necessary, a post-fabrication annealing treatment of the devices was performed in a N_2-filled glovebox (O_2 and H_2O within few ppm) on a Stuart SD 300 (600 W) hot plate. The ITO patterned glass substrate was purchased from Naranjo BV (thickness: ~100 nm). The materials (oxides, organic compounds, metals) were deposited by thermal sublimation in high vacuum ($< 5\cdot10^{-6}$ mBar) within a K.J. Lesker Spectros evaporating chamber. Al, MoO_3 and BCP were purchased from Sigma Aldrich and C_{70} from Lumtec (>99% grade). No additional purification was carried out. For each sample, three devices were obtained with a 0.18, 0.20 and 0.45 cm^2 photoactive area, respectively; no apparent influence of the device area on the device performances was observed.

The photovoltaic performances of the devices were determined in the glovebox from J-V profiles obtained with a Keithley 2400 source meter. The devices were exposed to AM 1.5 irradiation provided by a Sun 2000 solar simulator (ABET Tech.). 1 sun (100 mW cm^{-2}) irradiation was calibrated using a reference Si solar cell (Rera System).

Table S4. Photovoltaic parameters of TPA-T-DCV-Ph-F:C_{70} vacuum processed OSCs.

Weight ratio D:A	Voc [V]	Jsc [mA cm^{-2}]	FF [%]	PCE [%]
1:1	1.00	7.62	45.4	3.46
1:1	0.99	7.76	46.4	3.56
1:1	1.00	8.06	45.6	3.68

Figure S9 shows the AFM images of evaporated TPA-T-DCV-Ph-F pristine films, evidencing a very flat topography consistently with a barely crystallinity of the material turning amorphous after annealing at 100°C. Additionally, the AFM image of solution processed blend film (Figure S10) also showed smooth surface.

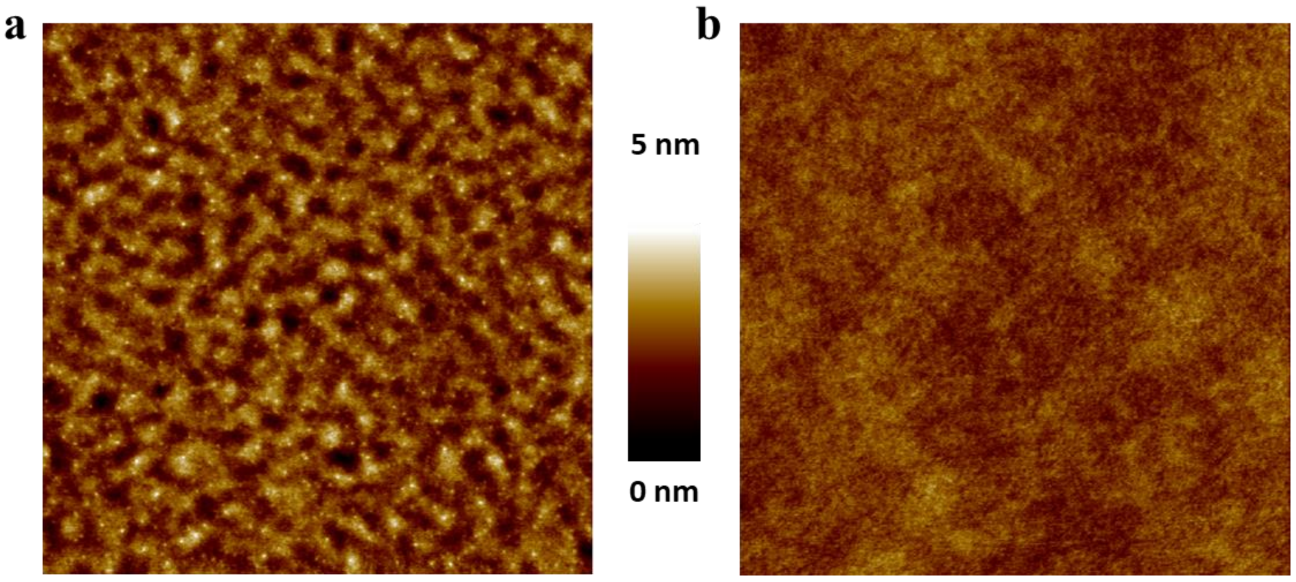

Fig. S9 4×4 µm² Height TM-AFM images captured on a 35 nm thick film of evaporated pristine TPA-T-DCV-Ph-F film. **(a)** RMS roughness: 0.552 nm, Z range: 5.39 nm, not annealed sample. **(b)** RMS roughness: 0.320 nm, Z range: 2.93 nm, annealed sample at 100°C during 10 min. The measurements were carried out using a Bruker Multimode 8 equipped with a Nanoscope V controller and with PPP-NCHR etched Si tip-probe manufactured by Nanosensors GmbH.

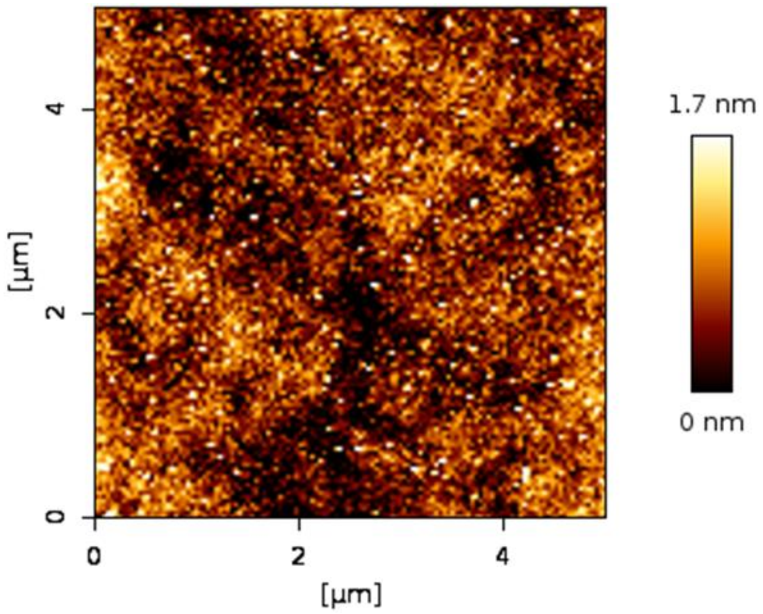

Fig. S10 AFM image of solution processed (TPA-T-DCV-Ph-F:PC70BM) blend with weight ratio of 1:2. RMS roughness: 0.387 nm.

References

1. Kozlov, O. V. *et al.* Simple donor-acceptor molecule with long exciton diffusion length for organic photovoltaics. *Org. Electron.* **53**, 185–190 (2018).

2. Barone, V. & Cossi, M. Quantum calculation of molecular energies and energy gradients in solution by a conductor solvent model. *J. Phys. Chem. A* **102**, 1995–2001 (1998).

3. Bässler, H. Charge Transport in Disordered Organic Photoconductors a Monte Carlo Simulation Study. *Phys. Status Solidi* **175**, 15–56 (1993).
